# 1441. LTBI screening cascade for non-US-born persons in a large health system assessed using EMR data

**DOI:** 10.1093/ofid/ofac492.1270

**Published:** 2022-12-15

**Authors:** Adrienne E Shapiro, Ayushi Gupta, Kristine F Lan, H Nina Kim

**Affiliations:** University of Washington and Fred Hutchinson Cancer Center, Seattle, Washington; University of Washington, Seattle, Washington; University of Washington, Seattle, Washington; University of Washington, Seattle, Washington

## Abstract

**Background:**

The majority of tuberculosis (TB) diagnosed in the US is reactivation TB in non-US-born (nUSb) persons. Guidelines recommend screening persons born in high TB burden countries for latent TB infection (LTBI) and treating if positive. We evaluated the LTBI cascade in primary care among nUSb persons in a large academic medical system in Washington State.

**Methods:**

We used electronic medical record (EMR) data to evaluate the LTBI screening cascade. nUSb individuals were identified using the primary language recorded in the EMR. Place of birth is not routinely collected in the EMR. All persons with a non-English primary language who entered care and attended one or more primary care visits in UW clinics between April 2016- April2021 were identified and considered eligible for LTBI screening. Persons with a documented IGRA or tuberculin skin test were considered screened for LTBI. Prescription records were reviewed to determine LTBI treatment.

**Results:**

5148 persons with non-English primary language attended primary care visits 2016-2021 and considered eligible for LTBI screening. Eligible persons were 58.5% female, median age 41 (IQR 30-58). Primary WHO regions of origin were Asia (37%), the Americas (36%), and Africa (19%), with a minority from North Africa, Middle East, and Europe. 1012 (20%) had a documented history of LTBI testing (Quantiferon-GOLD (N=949) or tuberculin skin testing (N=63)). Among 296 (29%) persons with a positive test for LTBI, 140 (47%) were treated for LTBI. The highest proportion of persons tested for LTBI were patients attending the HIV clinic (120, 66%) and the International/Refugee clinic (227, 81%). The majority of eligible patients were seen outside these clinics (N=4687) in whom the percent of persons tested ranged from 3-18% (14% overall).

LTBI cascade among non-US-born primary care patients

**Figure:**
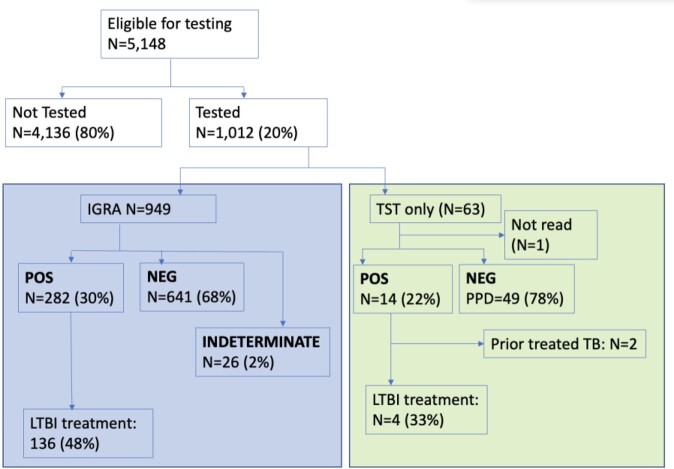

IGRA: interferon-gamma release assay; TST: tuberculin skin test.

**Conclusion:**

Although this subset underestimates the number of non-US-born individuals (since many non-US-born individuals use English as their primary language) eligible for screening, it nonetheless highlights the significant gap between actual and guideline-recommended screening in primary care, as well as further drop-off on the cascade from positive LTBI test to LTBI treatment. EMR platforms could be leveraged to prompt LTBI screening and treatment initiations in primary care to help achieve TB elimination goals in the US.

**Disclosures:**

**Adrienne E. Shapiro, MD, PhD**, Vir Biotechnology: Support as a trial site paid to my institution and non-financial support for medical writing GlaxoSmithKline third party funding to Vir support **H. Nina Kim, MD, MSc**, Gilead Sciences: Grant/Research Support.

